# Treatment Outcomes of Treatment-Naïve Hepatitis C Patients Co-Infected with HIV: A Systematic Review and Meta-Analysis of Observational Cohorts

**DOI:** 10.1371/journal.pone.0055373

**Published:** 2013-02-05

**Authors:** Anna Davies, Kasha P. Singh, Zara Shubber, Philipp duCros, Edward J. Mills, Graham Cooke, Nathan Ford

**Affiliations:** 1 Department of Infectious Diseases, Faculty of Medicine, Imperial College, London, United Kingdom; 2 Department of Infectious Diseases, Monash University, Melbourne, Victoria, Australia; 3 Division of Infection and Immunity, University College Hospital, London, United Kingdom; 4 Department of Infectious Disease Epidemiology, Faculty of Medicine, Imperial College, London, United Kingdom; 5 Manson Unit, Médecins Sans Frontières, London, United Kingdom; 6 Faculty of Health Sciences, University of Ottawa, Ottawa, Ontario, Canada; 7 Centre for Infectious Disease Epidemiology and Research, University of Cape Town, Cape Town, South Africa; University of North Carolina School of Medicine, United States of America

## Abstract

**Introduction:**

Co-infection with Hepatitis C (HCV) and HIV is common and HIV accelerates hepatic disease progression due to HCV. However, access to HCV treatment is limited and success rates are generally poor.

**Methods:**

We conducted a systematic review and meta-analysis to assess HCV treatment outcomes in observational cohorts. Two databases (Medline and EMBASE) were searched using a compound search strategy for cohort studies reporting HCV treatment outcomes (as determined by a sustained virological response, SVR) in HIV-positive patients initiating HCV treatment for the first time.

**Results:**

40 studies were included for review, providing outcomes on 5339 patients from 17 countries. The pooled proportion of patients achieving SVR was 38%. Significantly poorer outcomes were observed for patients infected with HCV genotypes 1 or 4 (pooled SVR 24.5%), compared to genotypes 2 or 3 (pooled SVR 59.8%). The pooled proportion of patients who discontinued treatment due to drug toxicities (reported by 33 studies) was low, at 4.3% (3.3–5.3%). Defaulting from treatment, reported by 33 studies, was also low (5.1%, 3.5–6.6%), as was on-treatment mortality (35 studies, 0.1% (0–0.2%)).

**Conclusions:**

These results, reported under programmatic conditions, are comparable to those reported in randomised clinical trials, and show that although HCV treatment outcomes are generally poor in HIV co-infected patients, those infected with HCV genotypes 2 or 3 have outcomes comparable to HIV-negative patients.

## Introduction

Co-infection with Hepatitis C (HCV) and HIV is common, and HIV accelerates hepatic disease progression due to HCV [1]. As a result, HCV has become a leading cause of death of people living with HIV in Western settings [2]. Successful treatment of HCV can improve hepatic fibrosis, reduce incidence of hepatocellular carcinoma, reduce mortality [3], [4], and has the potential to reduce disease transmission [5]. However, a number of factors contribute to the limited access to treatment for most of those infected globally: a long duration of therapy with a relatively complex system of treatment delivery, high drug costs, high toxicity of treatment and, perhaps most importantly, relatively poor success rates for HCV treatment in HIV/HCV co-infection.

A recent systematic review of clinical trials assessing HCV treatment outcomes in HIV co-infected patients reported that around 37% of patients achieve a sustained virological response (SVR) with pegylated interferon and ribavarin, with a lower success rate observed in patients infected with HCV genotypes 1 and 4 [6]. These outcomes are poorer than those seen in HIV negative patients [7]. Although clinical trials are appropriate for determining drug efficacy, outcomes may differ under programmatic conditions where adherence to treatment, patient and provider motivation and available resources may be limited [8]. We conducted a systematic review to assess the outcomes of HCV treatment in HIV co-infected patients in programmatic settings.

## Methods

### Search Strategy and Study Selection

Our systematic review was conducted in accordance with the criteria of the Preferred Reporting Items for Systematic Reviews and Meta-Analyses group [9]. Using a pre-defined protocol (File S1) Medline and EMBASE were systematically searched from inception to 05 May, 2012 using a compound search strategy. The initial title screen was conducted by one of us (AD) with full text articles reviewed in duplicate (AD, NF). The bibliographies of relevant articles were also hand searched for potentially relevant articles. Agreement on inclusion of final articles was made through consensus by the same reviewers. No language or geographical restriction was applied during the search, but only English language publications were included in the final review.

All cohort studies that reported treatment outcomes for in HIV-positive patients chronically infected with HCV and initiating HCV treatment for the first time were reviewed. Studies were excluded if they reported outcomes among patients with selected co-morbidities other than HIV, such as haemophilic or transplant patients, and if treatment outcomes involved acute HCV infection. Randomised trials were excluded in keeping with the aim of assessing outcomes in programmatic settings (defined as cohort reports in health care settings where there was no randomisation or control group comparison). In cases of potential duplication of studies, the largest report covering the longest time period was included and authors were contacted for clarification.

Patient and study characteristics were extracted in duplicate (AD, KS), with third party arbitration in case of disagreement (NF). The primary outcome was the proportion of patients achieving a SVR, calculated on an ‘intent-to-treat’ basis with all patients starting treatment contributing to the denominator. Secondary outcomes included the proportion of patients achieving a rapid virological response (RVR), defined as an undetectable (<50 copies/mL) serum level of HCV RNA at week 4 of treatment; discontinuation of treatment due to adverse drug reactions; loss to care (default); and death.

### Data Analysis

Point estimates and 95% confidence intervals (95% CI) were calculated for all primary and secondary outcomes. The variance of raw proportions was stabilised using a Freeman-Tukey type arcsine square-root transformation [10] and proportions were then pooled using a DerSimonian and Laird random effects model [11]. We calculated the τ^2^ statistic using DerSimonian and Laird’s method of moments estimator [11] to assess between-study heterogeneity [12]. Sources of heterogeneity were explored through univariate subgroup analyses to assess the potential influence of baseline liver damage, genotype, type of HCV treatment and co-treatment with highly-active antiretroviral therapy (HAART). All analyses were conducted using Stata version 12 (StataCorp LP, College Station, Texas, USA), with a P-value ≤0.05 considered as significant.

## Results

**Figure 1:**
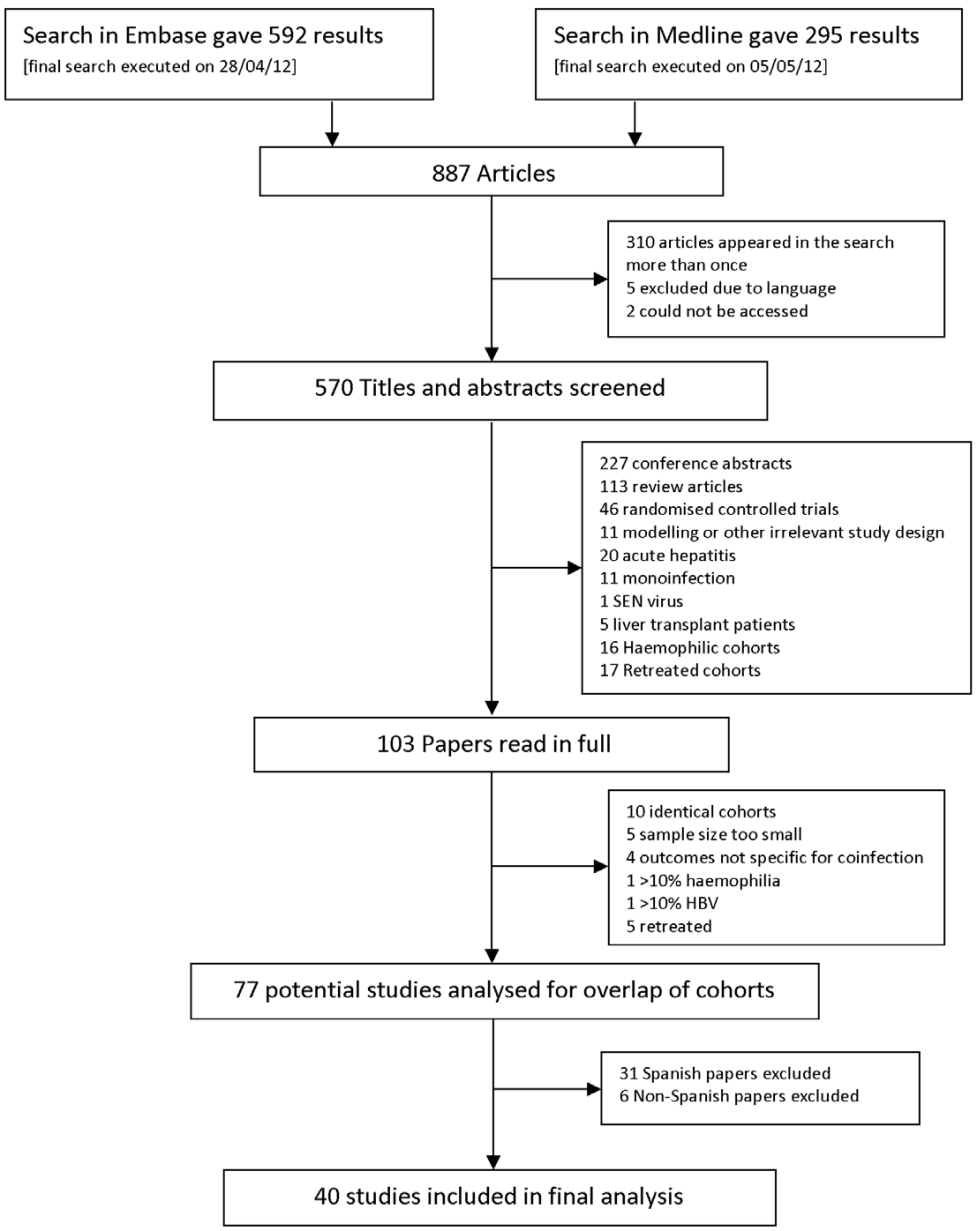
Identification of studies for inclusion.

887 articles were screened, and 103 of these were reviewed in full (figure 1). After identification of further papers which did not meet the inclusion criteria (e.g. studies that included retreated patients or studies that did not report treatment outcomes in full), we retained 77 studies for detailed review. Over half of these studies (37) were from Spain, and after correspondence with authors, 37 studies were excluded as partial or complete duplicate cohorts [13]–[52]. The final analysis included data on 5339 patients from 40 studies in 17 countries (Table 1).

**Table 1:** Characteristics of included studies.

Study	Study Characteristics			Patient Characteristics								
	Study design	Study setting	Sample size	Age	Risk factor for HCV acquisition	Genotype	Advanced liver damage at baseline	CD4 count at baseline (cells/µL)	Concurrent HAART	HCV treatment: pegylated (PEG) or standard (STD) interferon (IFN)	HCV treatment: fixed-dose (FD) or weight-based (WB) Ribavarin (RBV)	Duration of HCV treatment
Aguilar et al 2008	Prospective cohort	Italy	52	40 (37–42) Median (IQR)	NS	1/4∶55.8%; 2/3∶44.2%	NS	491 (411–620) Median (IQR)	21.2%	PEG-IFN	WB RBV	All 48 weeks
Amorosa et al 2010	Retrospective cohort	USA	212	48 (43–52) Median (IQR)	139 IVDU; 22 MSM; 87 WSM; 11 cocaine; 8 transfusion; 15 other	1/4∶87.3%; 2/3∶13.6%	32.5%	487 (355–675) Mean (IQR)	100%	PEG-IFN	WB RBV	All 48 weeks
Araujo et al 2011	Prospective cohort	Brazil	26	41 (32–56) Mean (range)	NS	1/4∶57.7%; 2/3∶42.3%	15.4%	570 (327–956) Mean (range)	69.2%	PEG-IFN	WB RBV	All 48 weeks
Avidan et al 2009	Prospective cohort	USA Spain and Austria	32	NS	14 IVDU; 18 MSM; 2 WSM	1/4∶82.1%; 2/3∶17.9%	17.9%	NS	84.4%	PEG-IFN	WB RBV	All 48 weeks
Berenguer et al 2011	Retrospective cohort	Spain	1701	41 (37–44) Median (IQR)	1382 IVDU; 75 excessive alcohol consumption	1/4∶62.9%; 2/3∶34.3%	38.2%	514 (390–720) Median (IQR)	88.3%	PEG-IFN	WB RBV	NS
Burbelo et al 2010	Prospective cohort	USA	29	NS	13 IVDU; 19 MSM	1/4∶82.8%; 2/3∶17.2%	NS	All <100	NS	PEG-IFN	WB RBV	All 48 weeks
Cesari et al 2009	Retrospective cohort	Italy	96	43 (41–46) Median (IQR)	NS	1/4∶50%; 2/3∶50%	17.7%	556 (422–722) Median (IQR)	90.6%	PEG-IFN	WB RBV	All 48 weeks
Cooper et al 2010	Retrospective cohort	Canada	41	NS	NS	1/4∶72.5%; 2/3∶27.5%	NS	549 (±274); Mean (SD)	82.9%	‘All formulations of IFN and RBV included’	NS	NS
Fleming et al 2005	Retrospective cohort	USA	21	NS	NS	1/4∶61.9%; 2/3∶38.1%	NS	NS	NS	NS	NS	NS
Gonvers et al 2010	Prospective cohort	Switzerland	47	NS	40 IVDU	1/4∶48.9%; 2/3∶51.1%	43.5%	NS	76.6%	PEG-IFN	WB RBV	Gen 1/4 = 48 weeks; Gen 2/3 = 24 weeks
James et al 2012	Retrospective cohort	Canada	21	46.6 Mean	9 IVDU; 15 MSM; 5 WSM; 9 blood products; 5 prisoners	1/4∶52.4%; 2/3∶47.6%	47.1%	556 Mean	71.4%	PEG-IFN	WB RBV	Gen 1 = 48 weeks; Gen 2/3 = 24, 32, 36 or 48 weeks according to viral response
Karlstrom et al 2008	Prospective cohort	Sweden	13	51 (38–62) Mean (range)	NS	2/3∶100%	NS	430 (250–800) Median (range)	76.9%	PEG-IFN	WB RBV	All 24 weeks
Kieran et al 2011	Retrospective cohort	Ireland	107	40 (23–58) Median (range)	67 IVDUs; 20 blood products; 14 sexual	1/4∶51.4%; 2/3∶48.6%	13.2%	5 patients <200	71.9%	PEG-IFN	WB RBV	NS
Laufer et al 2011	Prospective cohort	Argentina	20	40.5 (±4.8) Mean (SD)	NS	1/4∶100%	50%	All >200; 521 (±218) Mean (SD)	90%	PEG-IFN	WB RBV	All 48 weeks
Lerias de Almeida et al 2010	Retrospective cohort	Brazil	59	42 (±9) Mean (SD)	NS	NS	67.3%	432 Mean	NS	PEG-IFN	WB RBV	All 48 weeks
Lopez-Cortes et al 2012	Prospective cohort	Spain	58	44 (27–57) Median (range)	47 IVDU	2/3∶100%	42.9%	395 (92–1500) Median (range)	87.9%	PEG-IFN	FD RBV	Continue 20 weeks after undetectable serum RNA-HCV
Macias et al 2010	Prospective cohort	Spain	97	42 (40–45) Median (range)	87 IVDU	1/4∶68.0%; 2/3∶32%	78.5%	NS	95.9%	PEG-IFN	WB RBV	Gen 1 or 4 = 48 or 72 weeks; Gen 2 or 3 = 24 or 48 weeks
Marchetti et al 2012	Retrospective cohort	Italy	98	44 (41–46) Median (IQR)	87 IVDU; 3 MSM; 8 WSM	1/4∶45.9%; 2/3∶54.1%	74.5%	430 (321.5–567); Median (IQR)	98%	PEG-IFN plus RBV	WB RBV	48 or 72 weeks, ‘according to genotype’
Maru et al 2008	Retrospective cohort	USA	19	NS	19 prisoners	1/4∶78.9%; 2/3∶21.1%	NS	584 (490–696); Median (IQR)	79%	PEG-IFN	Mix of WB and FD RBV	NS
Mehta et al 2006	Retrospective cohort	USA	29	39 (36–43) Median (IQR)	19 IVDU	1/4∶89.7%; 2/3∶10.3%	52.9%	<200 = 2; 200–350 = 15; >350 = 12	58.6%	STD or PEG-IFN	± RBV (dosing NS)	NS
Michielsen et al 2009	Prospective cohort	Belgium	37	34 (17–60) Median (range)	15 IVDU; 7 blood products; 15 other or unknown	1/4∶59.5%; 2/3∶40.5%	55.9%	481 (222–1169); Median (range)	64.9%	PEG-IFN	WB RBV	All 52 weeks
Mira et al 2009	Prospective cohort	Spain	542	NS	462 IVDU	1/4∶65%; 2/3∶35%	68%	CD4≤250 = 39 patients; CD4>250 = 503 patients	82.7%	PEG-IFN	WB RBV	Gen 1 or 4 = 48 weeks; Gen 2 or 3 = 24 or 48 weeks
Murray et al 2011	Retrospective cohort	Canada	64	44 (39–50) Median (IQR)	33 IVDU; 27 MSM	1/4∶51.5%; 2/3∶48.5%	52%	400 (270–510) Median (IQR)	71.9%	PEG-IFN	Mix of WB and FD RBV	Gen 1 = 48 weeks; Gen 2/3 = 24 weeks (with potential to continue)
Nasti et al 2001	Prospective cohort	Italy	17	36 (27–47) Mean (range)	17 IVDU	1/4∶64.8%; 2/3∶35.2%	NS	445 (144) Mean (SD)	94.1%	STD-IFN	WB RBV	All 24 weeks
Neukam et al 2012	Prospective cohort	Spain and Germany	521	42 (39–46) Median (IQR)	391 IVDU	1/4∶70%; 2/3∶30%	39.5%	483 (355–665) Median (IQR)	–	PEG-IFN	WB RBV	Gen 1 or 4 = 48 or 72 weeks; Gen 2 or 3 = 24 weeks (when RVR achieved)
Nicot et al 2008	Retrospective cohort	France	35	41 (±8) Mean (SD)	NS	1/4∶60%; 2/3∶40%	NS	444 Mean	68.6%	PEG-IFN	WB RBV	All 48 weeks
Nischalke et al 2010	Prospective cohort	Germany	109	45 (29–68) Mean (range)	NS	NS	NS	524 (216–1902) Mean (range)	NS	PEG-IFN	RBV ‘according to current guidelines’	24 or 48 weeks ‘according to current guidelines’
Poizot-Martin et al 2003	Prospective cohort	France	62	36 (34–40) Median (IQR)	49 IVDU; 13 other	1/4∶67.7%; 2/3∶32.3%	76.7%	494 (327–657) Median (IQR)	88.7%	PEG or STD IFN	FD RBV	At least 24 weeks and up to 48 weeks
Reiberger et al 2011	Retrospective cohort	Germany and Austria	416	43 (±8) Mean (SD)	201 IVDU; 83 MSM; 20 WSM; 21 blood products; 91 unknown	1/4∶71.8%; 2/3∶28.2%	35.1%	530 (±242) Mean (SD)	56.9%	PEG-IFN	FD RBV (adjusted for genotype but not weight)	All 48 weeks
Reiberger et al 2008	Retrospective cohort	Austria	30	37 (±8) Mean (SD)	NS	1/4∶73.3%; 2/3∶26.7%	50%	568 (±276) Mean (SD)	60%	PEG-IFN	FD RBV (adjusted for genotype but not weight)	All 48 weeks (with option to extend to 72 weeks)
Righi et al 2008	Retrospective cohort	Italy	43	41 (±6.7) Mean (SD)	32 IVDU; 4 WSM	1/4∶48.8%; 2/3∶51.2%	40%	>500 in 22/43 patients; <350 in 6 patients	37.2%	PEG-IFN	WB RBV	Gen 1 or 4 = 48 weeks; Gen 3a = 24 weeks
Sacchi et al 2011	Prospective cohort	Italy	19	NS	NS	1/4∶42.1%; 2/3∶57.9%	18.2%	458 (122–842); Median (range)	HAART suspended during HCV treatment	PEG-IFN	WB RBV	All 48 weeks
Santin et al 2006	Prospective cohort	Spain	60	38.1±5.3 Mean (SD)	50 IVDU; 8 sexual	1/4∶68.3%; 2/3∶31.7%	NS	645 (±351) Mean (SD)	90%	PEG-IFN	WB RBV	Gen 1 or 4 = 48 weeks; Gen 2 or 3 = 24 weeks
Sarmento-Castro et al 2007	Prospective cohort	Portugal	53	32.6 Mean	45 IVDU; 8 sexual	1/4∶52.8%; 2/3∶47.2%	12.5%	585 Mean	69.8%	PEG-IFN	WB RBV	Gen 1 or 4 = 48 weeks; Gen 2 or 3 = 24 weeks
Taylor et al 2011	Prospective cohort	USA	11	46 (37–61) Mean (range)	All patients were recovering IVDU on methadone	1/4∶100%	54.5%	498 (210–868) Mean (range)	90.9%	PEG-IFN	WB RBV	All 48 weeks
Thein et al 2007	Prospective cohort	Australia	15	38.9 (±7.8) Mean (SD)	NS	1/4∶40%; 2/3∶60%	21.4%	363 (328–612); Mean (IQR)	33.3%	PEG-IFN	WB RBV	Gen 1 = 48 weeks; Gen 2 or 3 = 24 weeks (with option to extend)
Van den Eynde et al 2010	Retrospective cohort	Spain	278	39.8 (36.4–42.8) Median (range)	236 IVDU	1/4∶100%	62.3%	495 (357–692)	88.8%	PEG-IFN	Mix of WB and FD RBV	All 48 weeks
Wagner et al 2011	Retrospective cohort	USA	72	48. 1 Mean	NS	1/4∶70.8%; 2/3∶29.2%	NS	534 (±234); Mean (SD)	91.7%	PEG-IFN	RBV NS	24–32, 48 or 72 weeks
Yotsuyanagi et al 2009	Retrospective cohort	Japan	60	NS	High proportion blood products	1/4∶36.7%; 2/3∶30%	NS	NS	NS	STD-IFN	RBV given in 35 patients, not in 25 (dose not stated)	Gen 1 or 4 or other = 48 weeks; Gen 2 or 3 = 24 weeks
Zinkernagel et al 2006	Retrospective cohort	Switzerland	160	41 (37–44) Median (IQR)	122 IVDU; 21 WSM; 11 MSM; 6 blood products	1/4∶44.1%; 2/3∶54.4%	46.6%	490 (334–662.5) Median (IQR)	75.6%	PEG-IFN	FD RBV (dose adjusted for phenotype but not weight)	NS

FD RBV, fixed-dose ribavarin; IQR, interquartile range; IVDU, intravenous drug use; MSM, men who have sex with men; NS, not stated; PEG-IFN, pegylated interferon; SD, standard deviation; STD-IFN, standard interferon; WB RBV, weight-based ribavarin; WSM, women who have sex with men.

The proportion of patients with liver damage at baseline ranged from 12.5% to 74%. The majority of studies (36) included a mix of HCV genotypes. Three studies (from Argentina, Spain and the USA) were exclusively comprised of patients infected with genotypes 1 and 4 and two studies (from Sweden and Spain) were exclusively comprised of patients infected with genotypes 2 and 3.

HCV treatment comprised pegylated interferon and weight-based ribavarin in most cases, and the majority of patients (84%) received concomitant antiretroviral therapy. Liver damage was assessed by biopsy in over half (25) of studies. One study used fibroscan to assess liver damage, and 3 studies used a combination of the 2 techniques. Nine studies did not assess liver damage while the remainder of the studies (3) did not state the method used.

**Figure 2:**
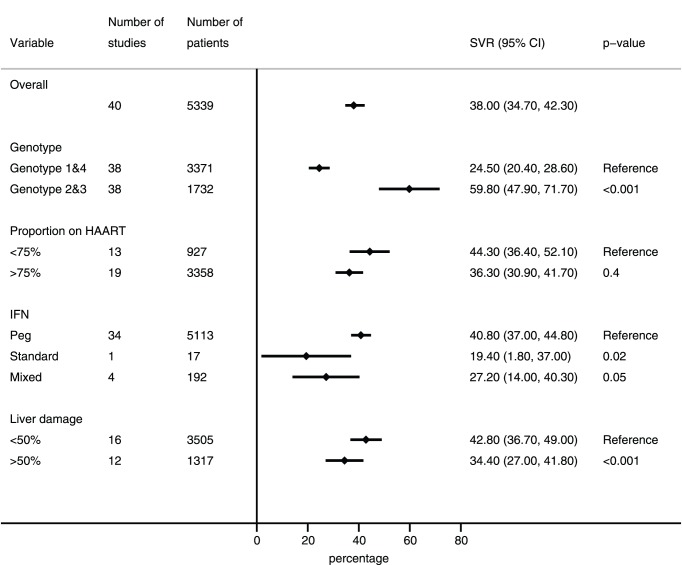
Sustained virological response (SVR) in patients co-infected with HCV and HIV by disease, patient and treatment covariates.

The proportion of patients achieving SVR ranged from 13.8% (2.2–32.9%) to 71.9% (48.2–90.5%), with a pooled proportion of 38% (34.7–42.3%) (τ^2^ 0.037). Three studies were ‘adherent cohorts’ comprising only patients who completed treatment; removing these studies from the analysis did not affect the overall result. The result was also unaffected by a sensitivity analysis that included all studies from Spain regardless of potential overlap (pooled SVR 39%). The most important determinant of treatment success was HCV genotype, with significantly poorer outcomes for patients infected with HCV genotypes 1 or 4 (3371 patients, pooled SVR 24.5% (95%CI 20.4–28.6%), compared to genotypes 2 or 3 (1878 patients, pooled SVR 59.8% (95%CI 47.9–71.7%). Cohorts in which more than 50% of patients had advanced liver fibrosis at baseline (Metavir F3 or F4 or equivalent) [53] had poorer outcomes compared to cohorts where less than 50% of patients had advanced liver disease (42.8%[36.7–49%] versus 34.4%[27–41.8%]). Subgroup analyses are summarized in Figure 2.

Rapid virological response, reported by 5 studies, was achieved by 30.9% of patients (11.2–50.8%). The pooled proportion of patients who discontinued treatment due to drug toxicities (reported by 33 studies) was low, at 4.3% (3.3–5.3%). Defaulting from treatment, reported by 33 studies, was also low (5.1%, 3.5–6.6%), as was on-treatment mortality, (35 studies, 0.1% (0–0.2%)).

## Discussion

Currently, access to effective HCV treatment is limited, particularly for those with HCV/HIV co-infection in resource-limited settings. This is reflected in this study by the paucity of data reoprted from such settings. Among the 40 studies assessed, only three were from resource-limited settings (two from Brazil and one from Argentina), and no reports were found from African countries, including Egypt where the burden of HCV is the highest in the world, or sub-Saharan Africa where the burden of HIV is the highest in the world. Limited access to treatment in resource-limited settings is in part due to the high cost of treatment, a perception of poorer outcomes of HCV treatment in HIV co-infected patients, and the potential difficulties associated with adherence and drug interactions under programmatic conditions.

Concern has recently been expressed that the relatively high efficacy of hepatitis treatment reported in clinical trials is not attained in subsequent effectiveness studies carried out in the general population under programmatic conditions [54]. In comparison to routine programmes, patients in clinical trials tend to be more adherent to treatment, and will usually receive treatment free of charge provided by highly motivated clinical staff working in optimal clinical settings [55]. Nevertheless, this review found that programmatic outcomes were in very close alignment to a systematic review of outcomes in clinical trials, which found that HCV treatment responses in HIV co-infected patients is lower than those observed in HIV-negative individuals [56]. Nevertheless, for HIV-positive patients infected with HCV genotypes 2 or 3, treatment outcomes are very similar (SVR 60%) to those reported for HIV-negative HCV patients infected with the same genotypes in programme settings (SVR 59%) [7].

Treatment completion was generally high, with few patients discontinuing treatment due to adverse events or defaulting from care. The use of HAART was not associated with better outcomes, which is consistent with other studies [57], [58].

We used a broad search strategy that allowed the inclusion of a large number of studies. We restricted studies to observational cohorts so that the expected outcomes would better reflect those observed in programmatic settings, but this can result in confounding. Concomitant use of medications, unreported mental or physical problems, or ancillary health service support could all influence treatment outcomes, but these factors were not reported and so could not be assessed. We attempted to use multivariate meta-regression to explore the potential influence of patient and programme level variables to explain differences in results between studies. However, this was restricted by inconsistent reporting between studies, so our exploration of associations was limited to univariate subgroup comparisons. In addition, bias may result from studies that pre-selected patients on the basis of characteristics that may influence treatment success, or excluded patients with risk factors for poor adherence. Furthermore, the final analysis only included studies published in English, which may lead to publication bias. Only five studies, however, were excluded on the basis of language and their influence would likely be small. Nevertheless, this review should be taken as an indication of outcomes and not as an exhaustive summary.

The treatment of HCV infection is likely to evolve rapidly as a result of a dynamic drug pipeline. For example, the first HCV protease inhibitors have just been recently approved. In the short to medium-term, however, the majority of HIV-positive patients living in resource-limited settings are unlikely to benefit from these newer treatments, just as they continue to lack access to many of the newer antiretroviral drugs for HIV that have been marketed in the West for many years. The results of this systematic review support the current practice of treatment in well resourced settings, whilst serving as a reminder for the need for better treatments. This review also highlights the need to encourage treatment of HCV/HIV co-infected patients in resource-limited settings to start programmes in parallel to efforts aimed at reducing costs of current treatment and gaining access to newer, interferon free regimens so that new advances in treatment can be rapidly accessed by all those that need them.

## Supporting Information

File S1 **Protocol.**
(PDF)

File S2 **PRISMA Checklist.**
(DOC)
