# Supplementary material for: Treatment Outcomes of Treatment-Naïve Hepatitis C Patients Co-Infected with HIV: A Systematic Review and Meta-Analysis of Observational Cohorts
Source: PLoS One. 2013 Feb 5;8(2):e55373. doi: 10.1371/journal.pone.0055373 (PMC3564801; doi:10.1371/journal.pone.0055373)
Supplement: File S1 — Protocol. (PDF) [file pone.0055373.s001.pdf]

Systematic review

Hepatitis C outcomes among HIV co-infected individuals in programmatic setting[1]

OBJECTIVE

To determine the programmatic outcomes for the treatment of hepatitis C virus in chronically infected, treatment naïve individuals who are co-infected with HIV.

METHODS

Search Strategy
- See annex

Databases
- MEDLINE
- EMBASE
- Bibliographies of relevant articles will be checked for further studies

Inclus**ions and exclusions**

Types of studies
- Prospective and retrospective cohorts reporting outcomes for ≥10 patients
- All other study designs, including randomized trials, will be excluded.

Types of participants
Inclusions:
- Chronic HCV-infected adults co-infected with HIV who have completed treatment for HCV.

Exclusions:
- Cohorts of patients with other co-morbidities (eg HBV, haemophilia, liver transplant). Cohorts in which >10% of patients have an additional co-morbidity will be excluded.
- Treatment-experienced cohorts. Cohorts in which <5% of patients are treatment-experienced will be included.

Types of interventions
- Interferon (either pegylated or standard) with or without ribavarin.

---

[1] Correspondence: Nathan Ford, Médecins Sans Frontières, 67-74 Saffron Hill, London EC1N 8QX. Email: nathan.ford@msf.org

Types of outcomes

Primary

- Sustained virological response (SVR), defined as undetectable serum levels of viral RNA 24 weeks following the end of treatment

Secondary

- Rapid virological response (RVR), defined as undetectable serum levels of viral RNA 24 weeks following the end of treatment
- Discontinuation of treatment due to adverse events
- Defaulting
- Mortality

DATA ANALYSIS

Point estimates and 95% confidence intervals (95% CI) will be calculated for all primary and secondary outcomes. The primary outcome of SVR will be determined in an 'intent-to-treat' like analysis, with all patients initiating HCV therapy contributing to the denominator. Proportions will be root-transformed, and estimates pooled by random effects meta-analysis.

Heterogeneity

The potential influence of clinical and programmatic covariates was explored through univariate subgroup analyses to assess the potential influence of:

- Genotype
- Type of HCV treatment
- Co-treatment with antiretroviral therapy
- Baseline liver damage

A P-value ≤0.05 considered as significant.

Statistical software

All analyses were conducted in Stata version 12

Annex: Search strategy

#22	#13 AND #18 AND #21
#21	#19 or #20
#20	SVR
#19	Sustained virological response
#18	#14 OR #15 OR #16 OR #17
#17	Intervention
#16	Therap*
#15	Treat*
#14	Explode "Therapeutics"[MeSH terms]
#13	#4 AND #12
#12	#5 OR #6 OR #7 OR #8 OR #9 OR #10 OR #11
#11	Acquired Immune Deficiency Syndrome
#10	Acquired Immunodeficiency Syndrome
#9	AIDS
#8	Human Immunodeficiency
#7	HIV
#6	Explode "HIV Infections"[MeSH Terms]
#5	Explode "HIV"[MeSH Terms]
#4	#1 OR #2 OR #3
#3	HCV
#2	Hepatitis C
#1	Explode "Hepatitis C"[MeSH Terms]
